# Longer or shorter? A large-scale randomized field experiment on the impact of free trial duration on sustainable user conversion in the Freemium model

**DOI:** 10.3389/fpsyg.2025.1568868

**Published:** 2025-06-18

**Authors:** Ling Zhang, Jiang Duan

**Affiliations:** 1 School of Computing and Artificial Intelligence, Southwestern University of Finance and Economics, Chengdu, China; 2 Chengdu Agricultural College, Chengdu, China

**Keywords:** Freemium model, free trial duration, user conversion, field experiment, behavioral operations

## Abstract

Trial duration is a critical strategic lever for improving subscription conversion rates on Software-as-a-Service (SaaS) platforms. While shorter trials in traditional subscription models can effectively boost immediate conversions during the trial period, the Freemium model—where users retain access to basic features after the trial—results in a large population of long-term active free users. Therefore, analyzing conversion in the Freemium context requires attention to both immediate and long-term dynamics. However, systematic empirical research on long-term user conversion under the Freemium model remains limited. This study, conducted in collaboration with a leading global SaaS company, implements a 2-year randomized field experiment involving 680,588 users across 190 countries (2023–2024). We develop a three-stage user conversion model to assess the impact of trial duration on behavior at each stage of the conversion funnel. The results show that extended trial periods significantly increase free trial adoption (Stage 1) and delayed conversion (Stage 3), but have no statistically significant effect on immediate conversion (Stage 2). These findings reflect the interplay between enhanced learning effects and demand cannibalization. Further analyses reveal that users with longer trials respond more favorably to feature-based promotions (e.g., AIGC feature launch), whereas shorter-trial users exhibit greater sensitivity to price-based incentives. Additionally, product functionality and users' economic and cultural contexts moderate the effect of trial duration on conversion. These findings offer practical guidance for optimizing trial design, tailoring promotional strategies, and localizing Freemium-based growth strategies in global SaaS markets.

## 1 Introduction

The Freemium model has become a dominant monetization strategy for Software-as-a-Service (SaaS) platforms. By offering core functionalities for free while restricting advanced features to paid subscription plans, this model effectively lowers user entry barriers and facilitates large-scale user acquisition and market penetration. However, converting free-tier users into paying subscribers remains a central challenge.

To improve conversion efficiency, many SaaS platforms implement time-limited free trials that allow users to experience premium features without upfront payment. In this context, trial duration emerges as a critical variable that directly influences customer acquisition cost and monetization efficiency. Longer trials can enhance user trust and familiarity through learning effects, but may also trigger free-riding behaviors and delayed conversion. In contrast, shorter trials can generate urgency and prompt quick decisions but may limit users' understanding of product value (Fader and Hardie, 2007).

Although prior studies have explored the trade-offs in trial strategies from both theoretical and empirical perspectives (Cheng and Liu, 2012; Dey et al., 2013; Foubert and Gijsbrechts, 2016; Sunada, 2018), most focus on immediate conversion within traditional subscription models. The specific stage-wise effects of trial duration in Freemium settings remain underexplored. While recent research has shown that shorter trials can improve short-term subscription rates (Yoganarasimhan et al., 2023), limited attention has been paid to reactivation behaviors during the delayed conversion stage and the dynamics of long-term retention following the end of the trial period.

Importantly, the Freemium model differs fundamentally from traditional subscription models in trial path design. In traditional models, access to all features ends with the trial, concentrating conversion pressure within the trial window. In contrast, Freemium users retain access to basic features after the trial ends, extending the engagement path and enabling the platform to gradually activate payment intent through ongoing interaction. This non-decisive transition emphasizes the need to evaluate trial strategies across multiple behavioral stages–including trial adoption, immediate conversion, and delayed conversion–while accounting for user heterogeneity across cultures, product types, and economic contexts.

To address this research gap, we collaborated with a leading global image-editing SaaS provider to conduct a large-scale randomized field experiment spanning 2 years, involving 680,588 new users across 190 countries. The experiment compared a standard 3-day trial (control) with a 7-day trial (treatment), reflecting industry norms. Leveraging user-level behavioral tracking data and a Local Average Treatment Effect (LATE) framework, we developed a three-stage conversion model tailored to Freemium environments to evaluate the causal impact of trial duration.

The results reveal that extending the trial period significantly increases trial adoption (+11.098%) and delayed conversion (+42.36%), but has no significant effect on immediate conversion, suggesting an offsetting mechanism between learning benefits and demand cannibalization. Further analysis shows that trial duration interacts with promotional strategies: longer-trial users are more responsive to feature-based campaigns (e.g., AIGC product launch), while shorter-trial users are more sensitive to price discounts. These effects are further moderated by product characteristics–exploratory tools amplify cannibalization risk, whereas task-oriented tools promote retention and paid conversion.

Moreover, we find that trial effectiveness is significantly moderated by cultural and economic contexts. In high-individualism markets, longer trials enhance conversion through self-guided exploration; in high long-term orientation markets, excessively long trials may suppress payment intent due to strategic delay behaviors (De Mooij and Hofstede, 2002).

In summary, this study contributes to the literature by identifying the distributed behavioral effects of trial duration across conversion stages, uncovering key interaction mechanisms with promotion types and product attributes, and quantifying cross-market heterogeneity. The findings offer actionable implications for SaaS platforms to develop culturally adaptive, product-aligned, and promotion-aware trial strategies to drive long-term conversion in global markets.

## 2 Literature review

### 2.1 Free trial design and behavioral responses

Free trials have emerged as a widely adopted strategy in digital product marketing, particularly in the software and information services sectors. Prior studies have explored how free trials shape users' perceptions of product quality and influence their purchase decisions. For instance, Reza et al. (2021) examine the shift in perceived quality distribution before and after user exposure, highlighting the joint impact of free content volume and pricing strategy. Cheng et al. (2015) focus on the cannibalization risks of software trials and propose optimization frameworks that balance trial generosity and revenue. Foubert and Gijsbrechts (2016) demonstrate that trial timing and user intensity are critical to effectiveness, using empirical data on interactive digital TV services. Meanwhile, Lin et al. (2019) find that free trial participation improves product reviews, thus contributing to downstream word-of-mouth dynamics.

More recent work also emphasizes the signaling role of trials. Wang and Özkan-Seely (2018) conceptualize trial duration as a quality signal when used in tandem with price, while Wang et al. (2023) explore how Freemium trial designs in online games affect revenue through phased user incentives. Notably, Yoganarasimhan et al. (2023) provide one of the first large-scale field experiments comparing different trial durations, showing that shorter (7-day) trials outperform longer ones (14- or 30-day) in conversion rates. However, most of these studies focus narrowly on immediate subscription outcomes, overlooking other stages in the user conversion funnel.

### 2.2 Subscription mechanisms and multi-stage conversion pathways

The broader literature on subscription services provides additional insights into user decision-making dynamics. Researchers in operations and information systems have examined how subscription vs. pay-per-use models affect firm profitability and user behavior (Belavina et al., 2017; Cachon and Feldman, 2011; Randhawa and Kumar, 2008). Niculescu et al. (2012) highlight consumer heterogeneity and network effects in subscription-based IT markets, while Wang et al. (2019) and Wang et al. (2022) show how bundling and targeted engagement (e.g., email marketing) shape usage patterns and conversion rates.

Moreover, a growing stream of literature emphasizes the multi-stage nature of user engagement. Danaher et al. (2003) employ a two-stage loyalty model to distinguish between online and offline consumer behavior. Huang et al. (2021) investigate how early registration affects both short-term conversion and long-term repurchase likelihood. These studies underscore the need to evaluate user lifecycle value beyond the initial conversion decision. Our study extends this line of inquiry by adopting a three-stage model that includes trial adoption, immediate conversion, and delayed reactivation–an approach particularly suited to Freemium environments.

### 2.3 CLV optimization and promotion strategy in SaaS

Understanding customer lifetime value (CLV) is critical to the success of SaaS platforms, particularly in designing promotional strategies. Prior work reveals that while price-based promotions can boost short-term purchase intent, they may also heighten price sensitivity and diminish long-term loyalty (Mela et al., 1997; Nijs et al., 2001; Pauwels et al., 2002). In contrast, feature-based promotions–such as the rollout of new functionalities–are shown to enhance both conversion and retention by elevating product engagement and perceived value (Slotegraaf and Pauwels, 2008). The effectiveness of these strategies is not uniform across contexts. Cultural norms and economic environments significantly shape how users respond to marketing interventions (De Mooij and Hofstede, 2002). For example, Gvili and Levy (2023) demonstrate that cultural orientation moderates the impact of electronic word-of-mouth on trust and behavior, while Peña-Garćıa et al. (2020) show that consumers in emerging markets exhibit different purchase triggers from those in developed regions. Our study integrates these perspectives by showing how trial duration interacts with promotion type–users with longer trials are more responsive to feature-based campaigns (e.g., AIGC releases), whereas shorter-trial users are more price-sensitive. We also find that the effectiveness of trial length is moderated by product attributes and national culture: longer trials improve conversion in individualist markets but may hinder it in long-term-oriented cultures due to strategic procrastination.

### 2.4 Empirical strategy and practice-oriented contributions

Finally, this study contributes to the practice-oriented literature on service operations. A number of recent studies have emphasized the importance of real-world experimentation and data-driven optimization in domains such as customer retention (Li, 2022), delivery efficiency (Cui et al., 2020), and digital engagement (Pattabhiramaiah et al., 2022). Retana et al. (2016) demonstrate the impact of proactive education in cloud services through field experimentation. Building on this tradition, we partnered with a global SaaS platform to conduct a 2-year randomized controlled experiment involving 680,588 users across 190 countries. Using local average treatment effect (LATE) estimation and clickstream tracking, we quantify the stage-specific effects of trial duration on user conversion and promotion sensitivity. The firm's subsequent adoption of our recommendations yielded measurable business gains, validating the practical relevance of our findings.

## 3 Theoretical model and hypothesis development

**Figure 1:**
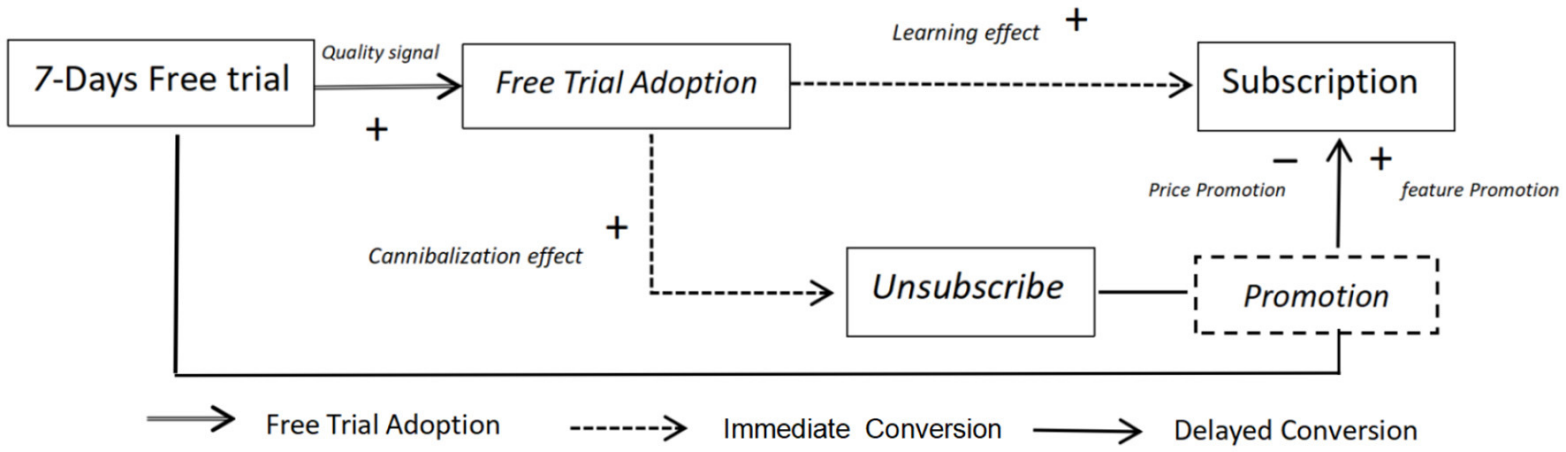
Research model. This figure illustrates the research model.

Within the context of the Freemium model, this study develops a three-stage user conversion framework, as illustrated in Figure 1.

**Stage 1: trial adoption** refers to the initial phase where new users accept an invitation to a free trial and complete the binding of their payment card information. The primary objective at this stage is to optimize the user experience to enhance engagement and increase the adoption rate of the trial offer.
**Stage 2: immediate conversion** occurs when users who have successfully bound their payment information during the trial period, and have not actively canceled, are automatically converted to paid subscribers at the conclusion of the trial. The conversion rate at this stage serves as a direct indicator of product attractiveness and user satisfaction.
**Stage 3: delayed conversion** targets users who did not convert immediately at the end of the trial. This stage seeks to reactivate potential demand through subsequent promotional efforts, such as price discounts or feature upgrades, thereby driving Delayed Conversion.

The model diagram employs three distinct types of arrows to represent the transition pathways between stages, while positive and negative signs indicate the hypothesized direction of effects on conversion outcomes at each stage. The subsequent sections provide a systematic empirical examination of these hypotheses and a detailed discussion of the underlying behavioral mechanisms.

### 3.1 Main effects of free trial duration on three-stage user conversion

#### 3.1.1 Free trial duration as a quality signal (H1a)

Free trials are an important mechanism for reducing users' perceived risk, particularly within the Freemium model. According to information asymmetry theory, when users lack sufficient information about product quality, firms can signal confidence by offering a longer trial period, thereby enhancing users' perceived reliability and value of the product (Foubert and Gijsbrechts, 2016; Reza et al., 2021; Wang and Özkan-Seely, 2018; Yoganarasimhan et al., 2023). A longer free trial is typically interpreted as a positive signal of product value, which increases users' willingness to engage and adopt the trial. Accordingly, we propose the following hypothesis:

**H1a**: Extending the free trial period significantly increases user conversion at Stage 1.

#### 3.1.2 Balancing learning effects and demand cannibalization (H1b)

While longer trial periods deepen user engagement and facilitate product learning and preference formation, they may also trigger a demand cannibalization effect: users fulfill their primary needs during the trial and reduce their willingness to pay afterward (Reza et al., 2021; Deng et al., 2023; Zhang et al., 2024). In Freemium contexts, where users can continue accessing basic functions without subscribing, this risk is further amplified. Thus, the positive and negative effects of trial extension on immediate conversion may offset each other. Based on this reasoning, we hypothesize:

**H1b**: Extending the free trial period has no significant effect on user conversion at Stage 2.

#### 3.1.3 Long-term interaction effects on delayed and overall conversion (H1c, H1d)

Research on customer lifecycle management highlights that early product interactions play a critical role in shaping subsequent payment behaviors (Slotegraaf and Pauwels, 2008; Huang et al., 2021). Extending the trial period can enhance users' familiarity with and attachment to the product, thereby increasing the likelihood of reactivating latent demand through subsequent promotional efforts, even if immediate conversion does not occur. Based on this reasoning, we propose the following hypothesis:

**H1c**: Extending the free trial period significantly enhances user conversion at Stage 3.

Moreover, by integrating outcomes from both immediate and delayed conversion stages, longer trial durations are expected to improve overall subscription rates and total platform revenue. Therefore, we propose:

**H1d**: Extending the free trial period leads to higher overall subscription rates and total revenue.

### 3.2 Moderating effects on the mechanisms of trial duration

#### 3.2.1 Moderating effect of promotion type (H2a)

Users' responses to promotional campaigns are shaped by their accumulated product knowledge during the trial. Users with shorter trial experiences are more sensitive to price discount promotions, which directly lower perceived purchase risks. In contrast, users with longer trial periods, who have formed a stronger understanding of product value, are more responsive to feature-based promotions that signal additional benefits (Pauwels et al., 2002; Mela et al., 1997; Slotegraaf and Pauwels, 2008). Therefore, we hypothesize:

**H2a**: Users with longer trial periods are more sensitive to feature-based promotions, while users with shorter trial periods are more sensitive to price discount promotions.

#### 3.2.2 Moderating effect of product functionality (H2b)

Product attributes may also moderate the effect of trial duration on conversion outcomes. Utilitarian features, centered on task completion and efficiency, support continuous usage needs. In contrast, creative features emphasize exploratory experiences, where user interest may exhibit a “novelty decay” phenomenon over time (Niu et al., 2023; Niu and Ma, 2025; Bossaerts et al., 2024). Hence, in products dominated by creative features, extending the trial period may reduce users' continued interest and subscription motivation. Accordingly, we propose:

**H2b**: In products dominated by creative features, extending the trial period may decrease users' subscription intentions.

#### 3.2.3 Moderating effect of economic and cultural backgrounds (H2c)

Economic development levels and cultural values significantly influence how users respond to trial strategies (De Mooij and Hofstede, 2002; Gvili and Levy, 2023; Peña-Garćıa et al., 2020). In high-Individualism markets, users are more inclined toward autonomous exploration, making trial extension more effective. Conversely, in high-Long-Term Orientation markets, users tend to delay decision-making, and excessively long trials may dampen subscription motivation. Therefore, we hypothesize:

**H2c**: In high-IDV markets, extending the free trial period enhances conversion rates; in high-LTO markets, extending the free trial period may suppress conversion rates.

## 4 Methodology

In this section, we commence by outlining the research design employed in this study. Then, we provide an overview of the data, followed by a detailed exposition of the model specification.

### 4.1 Randomized field experiment design and implementation

To investigate the causal impact of free trial duration on user conversion behavior, we employ a randomized field experiment following the methodological framework proposed by Levitt and List (2009). By randomly assigning users to treatment and control groups in real-world operational settings, this approach enables precise observation of behavioral changes and the collection of detailed user data in a natural context. Given its ability to effectively address endogeneity concerns and minimize estimation biases, randomized field experimentation is widely regarded as the gold standard for causal inference (Godinho de Matos and Adjerid, 2022).

This study was conducted in collaboration with a leading global SaaS platform specializing in image editing services. Operating under a Freemium model, the platform offers a mix of basic free functionalities and premium paid features, and serves ~400,000 daily active users–most of whom primarily engage with the free-tier services–thus providing an ideal setting for our field study.

To evaluate the causal effects of extending the free trial period from 3 to 7 days on user subscription behavior, we designed and implemented a large-scale randomized controlled experiment covering new users from nearly 190 countries. Prior to the experiment, newly registered users were offered a one-time free trial opportunity within their first week of joining the platform. By submitting their credit card information, users could activate a 3-day trial, which would automatically transition into a paid subscription unless manually canceled.

**Figure 2:**
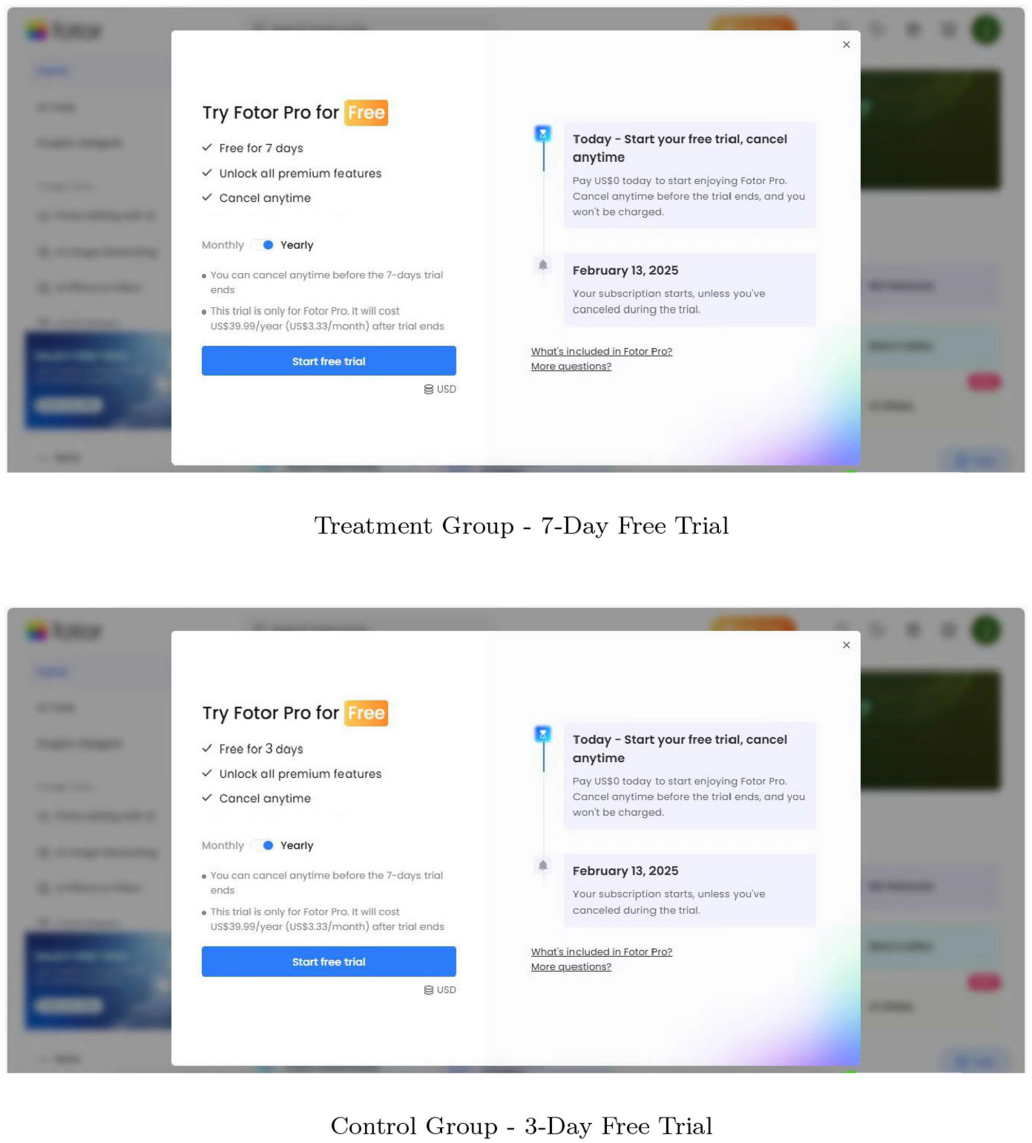
Free trial request page. This graph presents a comparison of the two groups for the product's free trial request page.

During the experiment, users were randomly assigned to either the treatment group (7-day free trial) or the control group (original 3-day trial). The design of the trial request page differed between the two groups, as illustrated in Figure 2, and the allocation ratio was determined based on a cost-benefit analysis to ensure both scientific rigor and operational feasibility.

Both groups experienced identical trial interactions: the system periodically displayed prompts encouraging users to explore premium features during the free trial. Users could cancel the auto-renewal at any time during the trial. If no cancellation occurred before the trial expiration, the subscription automatically transitioned to a paid plan according to the selected billing cycle (e.g., monthly or annually).

To ensure the validity of the results, a total of 680,588 new users who registered between July 18, 2022, and August 8, 2022, were randomly assigned to the treatment and control groups.^1^ The observation period lasted 2 years, concluding on July 15, 2024.

Additionally, we examined the impact of two key promotional phases on user conversion behavior: (1) a feature-based promotion following the launch of an AI-generated content (AIGC) functionality on November 18, 2022, lasting for 1 month, and (2) a price discount campaign offering a 20% discount from April 15 to April 28, 2023.

The randomized field experiment design adopted in this study ensures scientific rigor and robustness, providing strong empirical evidence for understanding the causal effects of free trial duration on user conversion in Freemium SaaS models and offering actionable insights for optimizing free trial strategies.

### 4.2 Data and variables

The dataset used in this study spans the period from July 18, 2022, to July 15, 2024, covering a total of 720 days. It includes a comprehensive range of user data, such as personal information (e.g., user ID, registration date, registration method, traffic sources and geographic location), transaction data (e.g., trial usage, subscription status, subscription type, cancellation records, and total expenditure), and behavioral data (e.g., page views, file imports/exports, downloads, and feature interactions). These data meticulously track user activities on the platform, recording precise timestamps, interaction points, and completion statuses, providing a solid foundation for the in-depth analysis of user behavior.

The high level of accuracy in the data enables a detailed portrayal of user payment behavior patterns, including trial periods, subscription types (annual or monthly), subscription expiration dates, and cancellation histories. In addition, the dataset.

### 4.3 Key variables and measures

To comprehensively analyze user conversion pathways within the Freemium model, this study categorizes key variables into four groups: treatment assignment, conversion outcomes, user behavior during the trial, and user background attributes. Table 1 provides detailed definitions.

**Table 1:** Key variable definitions.

**Variables**	**Definitions**
**Grouping variables**	**Treatment: A binary variable indicating whether a new user has completed the card binding process to participate in the free trial. A value of 1 signifies completion, while 0 signifies non-completion.^a^**

**Key outcome variables**	**Free trial adoption**: A binary variable indicating whether a user transitioned directly to a paid subscription upon the conclusion of the trial period. A value of 1 indicates successful conversion, while 0 indicates non-conversion.
	**Immediate conversion**: A binary variable indicating whether a user subscribes to a paid plan immediately after the free trial ends. A value of 1 denotes an immediate subscription, while a value of 0 indicates otherwise.
	**Delayed conversion**: A binary variable indicating whether users who did not convert during the trial period subsequently converted to a paid subscription through reactivation strategies. A value of 1 indicates successful conversion, while 0 indicates no conversion.
	**Overall subscription conversion (overall_Sub)**: A binary variable indicating whether a user successfully subscribed at least once during the 2-year observation period. A value of 1 denotes successful subscription, while 0 denotes non-subscription.
**Other outcome variables**	**Feature promotion subscription (FP_Sub)**: A binary variable indicating whether a free user successfully subscribed during the promotional period of a new feature launch. A value of 1 represents successful subscription, while 0 represents non-subscription.
	**Price promotion subscription (PP_Sub)**: A binary variable indicating whether a free user successfully subscribed during a price promotion period. A value of 1 represents successful subscription, while 0 represents non-subscription.
	**Cumulative spending (Cum_S)**: A continuous variable representing the total monetary amount spent on subscriptions by a user over the experimental observation period.
	**Cumulative annual subscriptions (CA_Sub)**: A discrete variable representing the total number of annual subscriptions accumulated by a user over the specified observation period.
	**Num of repeat subscriptions (Num_Re_Sub)**: A discrete variable representing the total number of Subscriptions accumulated by a user over the specified observation period.
**User behavior variables**	**VIP Experience(VIP_Ex)**: A binary variable indicating whether a user accessed VIP features during the free trial period. A value of 1 denotes access to VIP features, while 0 denotes no access.
	**VIP feature failure counts (VIP_FFC)**: A discrete variable representing the number of unsuccessful attempts to utilize VIP features during the user's free trial period.^b^
	**VIP task downloads (VIP_TDS)**: A discrete variable representing the total number of completed tasks that involved downloading content using VIP features during the user's free trial period.
**Feature attributes**	**Creative or task-oriented**: A binary variable indicating whether a feature is categorized as a creative feature. A value of 1 represents a creative feature, while 0 represents a task-oriented feature.^c^
**User attributes**	**GDP ranking**: A discrete variable representing the national-level per capita GDP ranking, sourced from the World Bank. A higher value indicates a lower rank, reflecting a weaker economic performance.
	**Cultural IDV**: An index measuring the extent to which a culture values individualism over collectivism. High-IDV cultures prioritize personal independence and self-expression, influencing users' product choices and decision-making (Hofstede, 1980)
	**Cultural LTO** : An index reflecting a culture's emphasis on long-term planning and future rewards. High-LTO cultures favor decisions that offer lasting benefits, influencing users' investments and goal-setting behaviors (Hofstede, 1980).^d^

^a^The experiment utilized a hash function to perform pseudo-random assignment based on unique user identifiers (e.g., user IDs, device IDs, IP addresses), ensuring fairness while maintaining data stability and traceability.

^b^*VIP Failure Experience*: Refers to reliability issues encountered by users when performing operations such as VIP account management, image editing, uploading, saving, rendering, and downloading. These failure events are defined by product managers and monitored and recorded by the system.

^c^Failure events include feature loading failures, crashes, permission issues, repeated unsuccessful operations, or inadequate functionality, which may negatively affect trial experiences and subsequent subscription decisions.

^d^Source: Hofstede Insights, retrieved from https://geerthofstede.com/research-and-vsm/dimension-data-matrix/, accessed on March 10, 2023.

First, treatment assignment distinguishes between users offered a 3-day vs. 7-day free trial. Conversion outcomes are measured across three critical stages: Free Trial Adoption (Stage 1), Immediate Conversion (Stage 2), and Delayed Conversion (Stage 3), as well as the Overall Subscription Conversion capturing any successful subscription within the 2-year observation window. Additional outcome variables include cumulative spending, repeat subscription behavior, and responsiveness to post-trial promotions (feature-based or price-based).

Second, user behavior variables capture engagement with premium features during the trial, such as VIP feature usage, task completion activities, and system-recorded failure experiences, providing insights into users' trial experiences and their relationship to conversion outcomes.

Finally, user background attributes–including GDP ranking and cultural dimensions (Individualism and Long-Term Orientation)–enable the analysis of heterogeneous responses across economic and cultural contexts. This multidimensional variable framework allows for a comprehensive investigation of the mechanisms and boundary conditions influencing trial-to-subscription behavior.

### 4.4 Model specification

To evaluate the impact of free trial duration strategies on user behavior, we utilize a binary variable to indicate whether user *i* has been assigned to the treatment group (7-day free trial). The model structure is as follows:


$$\begin{array}{l} {\text{Outcome}}_{i}=\beta_{0}+\beta_{1}{\text{Treatment}}_{i}+\gamma \cdot {\text{Controls}}_{i}+\epsilon_{i} \end{array}$$ (1)

In this model, *i* represents the user, and the binary variable Outcome covers various user transaction behaviors, such as whether the user adopted the free trial, whether they completed the trial conversion, whether they experienced delayed conversion, as well as VIP usage and feature clicks. When user *i* is assigned to the 7-day free trial group, the binary variable Treatment takes the value of 1.

Controls_*i*_ represents the vector of control variables, including user demographics, prior purchase history, and interaction frequency. γ is the corresponding vector of coefficients, reflecting the individual impact of each control variable on Outcome. β_0_ is the constant term, and ϵ_*i*_ is the error term that captures unobserved factors.

The coefficient β_1_ estimates the causal effect of assigning user *i* to the 7-day free trial group on user behaviors. Through this model, we can comprehensively analyze how the length of the free trial period affects user behavior, providing data-driven insights to optimize trial strategies, improve retention rates, and enhance overall business performance.

To further assess the moderating mechanism of user behavior and user characteristics on the free trial strategy, we introduce an interaction term. The model is as follows:


$$\begin{array}{l} {\text{Outcome}}_{i}=\beta_{0}+\beta_{1}{\text{Treatment}}_{i}+\beta_{2}{\text{Moderator}}_{i}+\beta_{3}({\text{Treatment}}_{i}\\ \;\times {\text{Moderator}}_{i})+\gamma \cdot {\text{Controls}}_{i}+\epsilon_{i} \end{array}$$ (2)

where Moderator represents the moderating variable, such as demographic characteristics or previous purchase behavior of users, to analyze how these characteristics influence the effectiveness of the free trial strategy. The coefficient β_3_ of the interaction term Treatment × Moderator is used to measure whether the effect of the moderating variable on user behavior changes depending on the free trial strategy.

## 5 Empirical results

We first conduct randomization checks between the groups to ensure there are no significant statistical differences. Then, we present the summary statistics to provide an overview of the data used in our study. These statistics offer a descriptive summary of the key variables and their distributions. Next, we present the estimation results of our model. Finally, we perform tests to delve deeper into the underlying mechanisms.

### 5.1 Randomization check and descriptive statistics

Randomization is an essential step in field experiments because it effectively reduces bias caused by confounding factors (Duflo et al., 2007). To ensure the success of the randomization, we conducted randomization checks on several variables: GDP Ranking, Cultural IDV, Cultural LTO, Registration Time, Registration Channel,^2^ and Traffic Sources.^3^ For these randomization checks, we performed analysis of variance (ANOVA), and the results showed no significant statistical differences in the covariates between the groups, as shown in Table 2. This indicates that there is no bias in the experimental setup, confirming the success of the randomization. This randomization process ensures that any differences in key outcomes can be attributed to variations in the trial duration, rather than other potential confounding factors.

**Table 2:** Randomization checks.

**Variable**	**Mean_control**	**Mean_treatment**	**Differences**	***T*-value**	***P*-value**
GDP ranking	84.6304	84.6577	–0.0273004	–0.2014	0.8404
Cultural IDV	40.15735	40.23082	–0.0734697	–1.0013	0.3167
Cultural LTO	42.53541	42.61061	–0.0752043	–1.1051	0.2691
Registration time	22859.20	22859.31	–0.112745	–0.6110	0.5412
Registration channels	1.395281	1.388690	0.0065913	1.1385	0.2549
Traffic sources	1.212788	1.209419	0.0033691	1.0266	0.1540

We report *t*-values and *p*-values for mean covariate differences between the treatment and control groups. The table displays raw statistics for GDP Ranking, Cultural IDV, and Cultural LTO. Registration Time is in time format, and Registration Channels and Traffic Sources are assigned numerical values for easier interpretation.

Table 3 summarizes key metrics for different conversion stages across the experimental group (7-day free trial), the control group (3-day free trial), and the overall sample. For each group and conversion stage, we report the sample size (*N*), percentage of the total, and mean conversion rates.

**Table 3:** Summary of trial and conversion data for control and treatment groups.

**Variable**	**Control group**	**Treatment group**	**Total**	**Increment**
**Free trial adoption**				
Number of observations (*N*)	479,317	201,271	680,588	–
Percent of total observations	70.4%	29.6%	100%	–
Number of free trial adoptions	4,101	1,914	6,015	–
Proportion of the total	68.18%	31.82%	100.0%	–
Free trial adoption rate	0.856%	0.951%	–	11.098%
**Immediate conversion**				
Number of immediate conversions	1,076	485	1,561	–
Proportion of the total	68.9%	31.1%	100%	–
Immediate conversion rate	0.224%	0.241%	–	7.342%
**Delayed conversion**				
Number of delayed conversions	688	412	1,100	–
Proportion of the total	62.5%	37.5%	100%	–
Delayed conversion rate	0.144%	0.205%	–	42.36%
**Total conversions**				
Number of total conversions	1,764	897	2,661	–
Proportion of the total	67.2%	32.8%	100%	–
Total conversion rate	0.368%	0.445%	–	20.92%

The treatment and control groups account for 29.6% and 70.4% of the total sample, respectively.^4^ The distribution of the sample is balanced, ensuring the representativeness and reliability of the results.

The average adoption rate for the free trial is 0.904%, indicating that a small proportion of users opted for the trial. However, the treatment group (7-day trial) had a significantly higher adoption rate of 0.951%, compared to 0.856% in the control group (3-day trial), a 11.098% increase, suggesting that extending the trial period increases user interest and participation.

In the immediate conversion stage, the average conversion rate was 0.233%. The treatment group showed a slightly higher conversion rate (0.241%) than the control group (0.224%), though the difference was small and not statistically significant. However, the delayed conversion stage showed a marked trend: the treatment group's delayed conversion rate was 0.205%, representing a 42.36% increase over the control group. This indicates that a longer trial period may better stimulate conversions in the later stages of the trial.

Finally, the overall conversion rate averaged 0.407%, with the treatment group showing a higher rate of 0.445% compared to the control group at 0.368%, a 20.92% increase. This further supports the positive effect of extending the trial period on user conversion rates. In sum, the extended trial period significantly enhanced user participation and delayed conversions, offering valuable insights for optimizing trial duration design.

### 5.2 Main results

#### 5.2.1 The impact of free trial duration on user conversion across three stages

We first analyze the effect of different free trial durations on User Conversion at various stages. Table 4 presents the regression results for the treatment's impact on Free Trial Adoption, Immediate Conversion, and Delayed Conversion.

**Table 4:** The impact of free trial duration on user conversion across three stages.

	**(1)**	**(2)**	**(3)**
**Variable**	**Free trial adoption**	**Immediate conversion**	**Delayed conversion**
Treatment	0.00095^***^	0.00016	0.00061^***^
	[0.00025]	[0.00013]	[0.00011]
Constant	0.00856^***^	0.00224^***^	0.00144^***^
	[0.00013]	[0.00007]	[0.00005]
*N*	680,588	680,588	680,588
Adj. *R*^2^	0.00002	0.00001	0.00005

Standard errors are shown in brackets. Significance levels are indicated as follows: ^*^p < 0.1, ^**^p < 0.05, ^***^p < 0.01.

In Column (1), the Treatment group (7-day free trial) shows a statistically significant positive effect on Free Trial Adoption (β = 0.00095, *p* < 0.01), indicating that the adoption rate is significantly higher in the 7-day trial group compared to the 3-day trial group, supporting Hypothesis 1a. In Column (3), the Treatment group also has a significant positive effect on Delayed Conversion (β = 0.00061, *p* < 0.01), suggesting that the 7-day trial promotes long-term conversions, which supports Hypothesis 1c.

These findings are statistically significant and economically meaningful. However, in Column (2), the effect of the 7-day free trial on the direct conversion from trial to paid subscription is positive but not statistically significant (β = 0.00016, *p*>0.1), which is consistent with Hypothesis 1b.

#### 5.2.2 Impact of free trial duration on overall subscription

We next explore the impact of different free trial durations on overall subscription rates over a 2-year observation period, focusing on the specific channels that drive revenue growth.

As shown in Table 5, the Treatment group demonstrates statistically significant positive effects on key variables, including Overall Subscription, Cumulative Spending, Number of Repeat Subscriptions, and Cumulative Annual Subscriptions. These results indicate that the Treatment group significantly boosts both user conversion and revenue growth, supporting Hypothesis 1d.

**Table 5:** Impact of free trial duration on overall subscription and revenue.

	**(1)**	**(2)**	**(3)**	**(4)**
**Variable**	**Overall_Sub**	**Cum_S**	**Num_ Re_Sub**	**CA_Sub**
Treatment	0.00079^***^	0.04645^***^	0.00796^***^	0.00227^***^
	[0.00017]	[0.01087]	[0.00064]	[0.00038]
Constant	0.00367^***^	0.17837^***^	0.00366^***^	0.01033^***^
	[0.00009]	[0.00524]	[0.00009]	[0.00019]
*N*	680,588	680,588	680,588	680,588
Adj. *R*^2^	0.00003	0.00002	0.00050	0.00005

Standard errors are shown in brackets. Significance levels are indicated as follows: ^*^p < 0.1, ^**^p < 0.05, ^***^p < 0.01.

Notably, the results for *Number of Repeat Subscriptions* in Column (3) and *Cumulative Annual Subscriptions* in Column (4) reveal the specific mechanisms through which revenue growth is achieved. On one hand, the Treatment group leads to a substantial increase in repeat purchases. On the other hand, a greater proportion of users opt for long-term subscription plans, such as annual subscriptions. These findings suggest that a longer free trial period enhances the learning effect, enabling users to better understand the product's features and value. As a result, users develop a stronger attachment to the product, leading to improved retention. Over time, this deeper engagement reinforces users' perception of the product's long-term value, motivating them to commit to longer subscription plans, thereby driving higher rates of repeat purchases and annual subscription conversions.

### 5.3 Exploring the underlying mechanisms

Existing empirical research has demonstrated that shorter trial periods can accelerate users' subscription decisions. The primary objective of a short trial period is to expedite users' decision-making processes, allowing them to quickly assess product value and transition to paid subscriptions, while minimizing cannibalization and free-riding behaviors. In this context, the experimental company initially adopted a 3-day VIP feature free trial to enhance conversion rates and stabilize cash flow.

However, some studies argue that longer trial periods may offer additional benefits, particularly by signaling higher product quality, fostering deeper user learning effects, and enhancing customer lifetime value (CLV). Longer trial periods are believed to provide stronger quality signals, facilitate more comprehensive product understanding, and subsequently increase user retention. As the Freemium model becomes increasingly widespread, this alternative perspective warrants further investigation.

In the present experiment, the company compared the effects of 7- and 3-day free trial periods. The results revealed that the 7-day trial significantly outperformed the 3-day trial in terms of both conversion rates and revenue generation, exceeding expectations. This finding contradicts the company's prior strategic assumptions and motivates a deeper exploration of the underlying mechanisms. While the positive impact of longer trials on *Free Trial Adoption* has been extensively documented in the literature on value expectation theory and quality signaling, this study focuses on analyzing how trial duration affects subsequent stages, namely *Immediate Conversion* and *Delayed Conversion*.

To investigate these mechanisms, we examine users' behavioral patterns during the trial period. Prior research suggests that trial engagement mediates the relationship between trial duration and conversion outcomes. Accordingly, we focus on several key behavioral indicators:*VIP experience*: indicates whether users engaged with VIP features during the trial and completed tasks satisfactorily. Successful participation improves perceived value of the product and increases purchase intentions. *VIP task downloads*: captures the number of tasks completed using VIP features. Frequent usage may reflect stronger product familiarity; however, extensive task completion could also lead to demand cannibalization, wherein users satisfy their needs during the trial and reduce their immediate subscription propensity. *VIP feature failure count*: measures the number of unsuccessful attempts to utilize VIP features. Negative experiences during the trial may erode user trust and diminish conversion likelihood. *VIP task downloads per day*: represents the daily frequency of VIP feature usage. Higher daily engagement generally correlates with greater reliance on premium features, potentially increasing the likelihood of subscription.

These behavioral variations are hypothesized to drive differences in subscription outcomes. To test these relationships, we first perform regression analyses examining the impact of trial duration on each behavioral variable (see Table 6). Subsequently, we explore how these behavioral patterns influence subscription decisions (see Tables 7, 8). Given the randomized assignment of trial durations, the study design supports a causal interpretation of the observed relationships.

**Table 6:** Impact of free trial duration on user behaviors.

	**(1)**	**(2)**	**(3)**	**(4)**
**Variable**	**VIP_ Ex**	**VIP_TDS**	**VIP_FFC**	**VIP_TDS/per day**
Treatment	0.00047^***^	0.00877^**^	0.00735^***^	0.00003
	[0.00009]	[0.00281]	[0.00128]	[0.00047]
Constant	0.00079^***^	0.00642^***^	0.02816^***^	0.00214^***^
	[0.00004]	[0.00081]	[0.00043]	[0.00026]
*N*	680,588	680,588	680,588	680,588
Adj. *R*^2^	0.00005	0.00002	0.00007	0.0000001

Standard errors are shown in brackets. Significance levels are indicated as follows: ^*^p < 0.1, ^**^p < 0.05, ^***^p < 0.01.

**Table 7:** The impact of user trial behaviors on immediate conversion.

**Variable**	**(1)**	**(2)**	**(3)**	**(4)**
	**Immediate**	**Immediate**	**Immediate**	**Immediate**
VIP_Ex	0.27018^***^			
	[0.02735]			
Treatment	–0.00015	0.00023^*^	0.00033^**^	0.00022^*^
	[0.00012]	[0.00013]	[0.00013]	[0.00013]
VIP_Ex × treatment	0.14526^***^			
	[0.04130]			
VIP_TDS		0.01397***		
		[0.00356]		
VIP_TDS × treatment		–0.01261^***^		
		[0.00362]		
VIP_FFC			0.00873***	
			[0.00186]	
VIP_FFC × treatment			–0.00644^***^	
			[0.00197]	
VIP_TDS/per day				0.04191^***^
				[0.00036]
VIP_TDS/per day × treatment				–0.03240^***^
				[0.00071]
Constant	0.00203^***^	0.00216^***^	0.00200^***^	0.00216^***^
	[0.00007]	[0.00007]	[0.00008]	[0.00007]
*N*	680,588	680,588	680,588	680,588
Adj. *R*^2^	0.0472	0.0194	0.0023	0.0194

Standard errors are shown in brackets. Significance levels are indicated as follows: ^*^p < 0.1, ^**^p < 0.05, ^***^p < 0.01.

**Table 8:** The impact of user trial behaviors on delayed conversion.

	**(1)**	**(2)**	**(3)**	**(4)**	**(5)**
**Variable**	**Delayed**	**Delayed**	**Delayed**	**Delayed**	**Delayed**
Free trial adoption	0.04061^***^				
	[0.00312]				
Treatment	0.00017^*^	0.00045^***^	0.00054^***^	0.00067^***^	0.00054^***^
	[0.00009]	[0.00011]	[0.00011]	[0.00012]	[0.00011]
Free trial adoption × treatment	0.04225^***^				
	[0.00707]				
VIP_Ex		0.06244^***^			
		[0.01265]			
VIP_Ex × treatment		0.10501^***^			
		[0.02672]			
VIP_TDS			0.00111^**^		
			[0.00056]		
VIP_TDS × treatment			0.00422^***^		
			[0.00133]		
VIP_FFC				0.00331***	
				[0.00111]	
VIP_FFC × treatment				–0.00230^**^	
				[0.00117]	
VIP_TDS/per day					0.00332^**^
					[0.0069]
VIP_TDS/per day × treatment					0.03394^***^
					[0.008604]
Constant	0.00109^***^	0.00139^***^	0.00143^***^	0.00134^***^	0.00143^***^
	[0.00005]	[0.00005]	[0.00005]	[0.00006]	[0.00005]
*N*	680,588	680,588	680,588	680,588	680,588
Adj. *R*^2^	0.01800	0.00806	0.00781	0.00121	0.00778

Standard errors are shown in brackets. Significance levels are indicated as follows: ^*^p < 0.1, ^**^p < 0.05, ^***^p < 0.01.

#### 5.3.1 The impact of trial duration on user behaviors during the trial period

Table 6 presents the effects of extended trial duration on user behaviors during the trial phase. In Column (1), extending the free trial period significantly increases the likelihood of users engaging with VIP features (β = 0.00047, *p* < 0.01), suggesting that a longer trial enhances users' perceived value of the product. In Column (2), a longer trial period also leads to a significant increase in the number of completed tasks using VIP features (β = 0.00877, *p* < 0.05), indicating a higher level of product utilization. However, Column (3) shows that extending the trial period significantly raises the count of VIP feature failures (β = 0.00735, *p* < 0.01), implying that although users have more opportunities for engagement, the increased likelihood of negative experiences may erode product trust and adversely affect conversion decisions. Column (4) reveals that the effect of trial duration on VIP task downloads per day is positive but not statistically significant (β = 0.00003, *p*>0.1), suggesting that a longer trial disperses user engagement more evenly across days rather than intensifying daily activity, potentially weakening the positive effect of usage intensity on conversion outcomes.

#### 5.3.2 The impact of user trial behaviors on immediate conversion

We then examine how user behaviors during the trial period affect immediate conversion outcomes. Table 7 reports the empirical results for interaction effects. In Column (1), the interaction between *VIP Experience* and Treatment (7-day trial group) is significantly positive (β = 0.14526, *p* < 0.01), indicating that users who successfully engaged with VIP features during the extended trial exhibited a higher immediate conversion rate. In contrast, Column (2) shows that the interaction between *VIP Task Downloads* and Treatment is significantly negative (β = −0.01261, *p* < 0.01), supporting the demand saturation hypothesis: excessive task completion during the trial fulfills users' primary needs and diminishes subsequent subscription willingness. Similarly, Column (3) finds that the interaction between *VIP Feature Failure Counts* and Treatment is also significantly negative (β = −0.00644, *p* < 0.01), suggesting that trial-period failure experiences reduce overall product evaluation and hinder immediate conversion, consistent with Loss Aversion Theory. While *VIP Task Downloads per Day* shows a positive effect on immediate conversion (β = 0.04191, *p* < 0.01), Column (4) reveals that its interaction with Treatment is significantly negative (β = −0.03240, *p* < 0.01), further corroborating the existence of demand saturation effects in longer trials. Taken together, the impact of trial duration on immediate conversion exhibits a dual nature: while positive feature experiences promote conversion, excessive task completion and negative experiences suppress it. These offsetting effects explain the empirical finding under Hypothesis 1b that extending the trial period has no significant effect on immediate conversion.

#### 5.3.3 The impact of user trial behaviors on delayed conversion

Further, Table 8 reports the effects of user behaviors during the trial period on long-term delayed conversion outcomes. In Column (2), the interaction between *VIP Experience* and Treatment is significantly positive (β = 0.10501, *p* < 0.01), indicating that positive feature experiences during the trial not only enhance immediate conversion but also have a sustained positive impact on delayed conversion. In Column (4), the interaction between *VIP Feature Failure Counts* and Treatment remains significantly negative (β = −0.00230, *p* < 0.05), suggesting that negative experiences during the trial continue to suppress delayed conversion outcomes. Interestingly, in contrast to the findings for immediate conversion, Column (3)shows that the interaction between *VIP Task Downloads* and Treatment is significantly positive (β = 0.00422, *p* < 0.01), and Column (5) reveals that the interaction between *VIP Task Downloads per Day* and Treatment is also significantly positive (β = 0.03394, *p* < 0.01).

These findings imply that although high-frequency usage during the trial may initially trigger demand saturation and reduce immediate conversion, over time, deeper product engagement strengthens users' perception of long-term product value, thereby stimulating latent demand and increasing the likelihood of delayed subscription. This pattern is consistent with behavioral economics theories emphasizing the trade-off between immediate gratification and long-term value realization. Specifically, users who engage more intensively with core functionalities during the trial may initially defer payment but gradually accumulate appreciation for the product's enduring value, ultimately leading to delayed subscription.

Overall, the extension of the trial period enhances positive user experiences and deepens product engagement, laying a solid foundation for long-term subscription conversion and providing empirical support for Hypothesis 1c.

#### 5.3.4 Differential impact of free trial duration on advertising promotions

**Figure 3:**
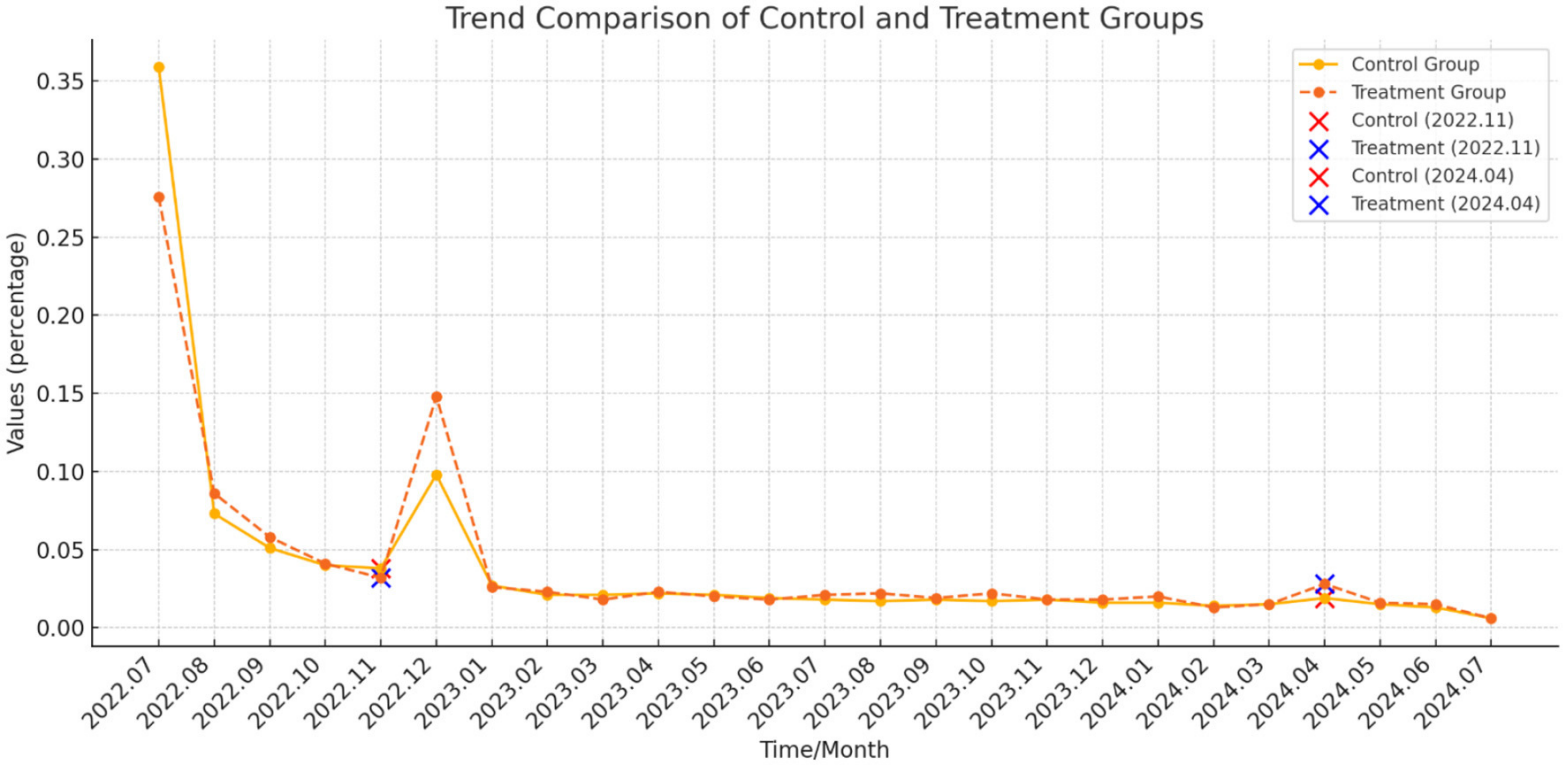
Dynamic evolution of subscription conversion rate. This graph presents the dynamic temporal trend in the percentage of monthly subscription conversions relative to the total conversions over the 2-year observation period for both user groups.

Figure 3 illustrates the dynamic evolution of monthly conversion rates over the 2-year observation period. During the experimental phase (July 18, 2022, to August 8, 2022), a substantial influx of new users joined the platform each day and became eligible for trial-based conversions. Throughout this period, the treatment group consistently exhibited a significantly higher trial adoption rate compared to the control group, as evidenced by the treatment group's curve (dashed line) remaining above that of the control group (solid line). However, as the experiment concluded, the gap between the two groups' conversion rates gradually narrowed, as reflected by the convergence of the dashed and solid lines.

Following the experiment, during the extended observation period, both groups entered the delayed conversion phase, as all users lost eligibility for trial-based conversions. At this stage, the overall conversion rates dropped sharply to a lower baseline. Notably, the treatment group's conversion rate progressively surpassed that of the control group, suggesting that trial behaviors during the experimental phase had a lasting effect on subsequent subscription outcomes.

Although the overall delayed conversion rate remained relatively low throughout the observation period, external interventions–particularly advertising promotions–emerged as key drivers of conversions among free users. Figure 3 highlights two major promotional events: the AIGC New Feature Launch Promotion (November 18, 2022, to December 18, 2022) and the 20% Discount Promotion (April 15, 2023, to April 28, 2023).

During these promotional periods, a noticeable increase in conversion rates was observed, with the treatment group experiencing a more pronounced uplift compared to the control group. This pattern suggests that trial duration shapes users' sensitivity to different promotional stimuli, thereby influencing their subsequent purchasing decisions.

To further examine these patterns, we conducted regression analyses controlling for prior subscription behavior–namely, Subscription Before Feature Promotion and Subscription Before Price Promotion. Table 9 reports the results.

**Table 9:** Differential impact of free trial duration on advertising promotions.

	**(1)**	**(2)**	**(3)**	**(4)**
**Variable**	**FP_Sub**	**PP_Sub**	**FP_Sub**	**PP_Sub**
Treatment	0.00794^***^	–0.00004^*^	0.00022^***^	–0.00001
	[0.00020]	[0.00003]	[0.00006]	[0.00002]
BFP_Sub	0.03275^***^		−0.37811^***^	
	[0.00259]		[0.02407]	
BPP_Sub		0.02219^***^		0.02308^***^
		[0.00302]		[0.00350]
Free trial adoption			0.39622^***^	–0.00051
			[0.02417]	[0.00147]
Free trial adoption × treatment			0.47542^***^	–0.00278^*^
			[0.02213]	[0.00167]
Constant	0.00006^***^	0.00006^***^	0.00042^***^	0.00006^***^
	[0.00002]	[0.00001]	[0.00002]	[0.00001]
*N*	680,588	680,588	680,588	680,588
Adj. *R*^2^	0.0076	0.0133	0.7172	0.0135

Standard errors are shown in brackets. Significance levels are indicated as follows: ^*^p < 0.1, ^**^p < 0.05, ^***^p < 0.01.

In Column (1), the treatment group shows a significantly positive effect on conversion during the feature promotion period (β = 0.00794, *p* < 0.01). In Column (2), however, the treatment group exhibits a marginally significant negative effect on conversion during the price promotion period (β = −0.00004, *p* < 0.1). Moreover, the interaction between treatment and *Free Trial Adoption* is stronger. Specifically, during the feature promotion, the interaction effect is significantly positive (β = 0.47542, *p* < 0.01), much larger than the main treatment effect (β = 0.00794, *p* < 0.01). In contrast, during the price promotion, the interaction effect is significantly negative (β = −0.00278, *p* < 0.1), more negative than the treatment effect alone (β = −0.00004, *p* < 0.1). These results suggest that users who participated in the 7-day free trial, particularly those who engaged with VIP features, are less sensitive to price promotions but more responsive to feature-based promotions compared to users in the 3-day group. This finding provides strong empirical support for Hypothesis 2a.

### 5.4 Moderating effect of feature creativity

Within the functional architecture of SaaS products, different modules can be categorized based on their inherent attributes, and users may exhibit distinct behavioral patterns during their exploration and learning processes. For example, utilitarian features primarily fulfill users' basic needs for work efficiency and task completion, typically aligning with users' instrumental expectations, while creative support features emphasize exploratory experiences and emotional engagement, offering higher perceived value. On the one hand, due to the higher perceived value of creative support features, users are more likely to interact with these features over a longer trial period, fostering stronger emotional connections and increasing conversion likelihood (Bossaerts et al., 2024). On the other hand, research by Niu et al. (2023) and Niu and Ma (2025) reveals an inverted U-shaped relationship between positively framed attributes and advertising effectiveness, suggesting that consumer interest in highly novel products typically follows an inverted U-shaped curve: excessively long trial periods may lead to a decline in users' interest in creative support features, ultimately affecting conversion intentions.

Building upon this theoretical foundation, the present study further investigates how different functional attributes moderate the effect of trial duration on user conversion pathways. The functionalities of the experimental product have been carefully classified, with product managers distinguishing between basic task-oriented functional modules and creativity-oriented functional modules (see Table 10). Creativity-oriented functions are characterized by their exploratory, unique, and entertainment-driven nature, emphasizing user creativity and personalized experiences. In contrast, basic task-oriented functions primarily address rigid user demands, prioritizing efficiency and stability in task execution. Under this classification framework, this study examines whether functional attributes moderate the impact of trial duration on user conversion pathways and explores the underlying mechanisms at play.

**Table 10:** Comparison between task-oriented tools and creative tools.

**Feature dimension**	**Task-oriented tools**	**Creative tools**
Uniqueness	Low uniqueness; similar functionalities across products, with competition primarily based on execution efficiency and accuracy.	Flexible demand, low frequency, emotionally and creatively driven.
Value proposition	Improve task efficiency and operational accuracy.	Meet personalized creation needs and enhance experiential value.
User behavior pattern	Functional decisions, task-driven.	Emotional decisions, inspired by creativity or inspiration.
Product positioning	Productivity tools	Creative tools
Theoretical foundation	Utility maximization theory	Experience economy theory

The data analysis results indicate that Treatment × Creativity exerts a significant effect on all stages of user conversion except for *Free Trial Adoption*, thereby confirming the crucial role of product functionality in moderating the influence of trial duration on user decision-making processes. Specifically, as shown in Column (1) of Table 11, the interaction term Treatment × Creativity is significantly positive for *Free Trial Adoption* (β = 0.76980, *p* < 0.01), suggesting that when a product's free trial includes creativity-oriented functional modules, extending the trial duration can effectively enhance user adoption rates.

**Table 11:** Moderating effect of feature creativity.

	**(1)**	**(2)**	**(3)**	**(4)**
**Variable**	**Free trial**	**Immediate**	**Delayed**	**Num of repeat**
Creativity	0.21184^***^	0.08252^***^	0.75299^***^	0.83553^***^
	[0.03816]	[0.02564]	[0.03963]	[0.03384]
Treatment	0.00222^***^	–0.00006	0.00048^***^	0.00681^***^
	[0.00022]	[0.00012]	[0.00011]	[0.00059]
Creativity × treatment	0.76980^***^	–0.00765	–0.65653^***^	-0.42434^***^
	[0.03839]	[0.02765]	[0.04129]	[0.07675]
Constant	0.00850^***^	0.00222^***^	0.00125^***^	0.00346^***^
	[0.00013]	[0.00007]	[0.00005]	[0.00008]
*N*	680,588	680,588	680,588	680,588
Adj. *R*^2^	0.1076	0.0029	0.0665	0.0114

Standard errors are shown in brackets. Significance levels are indicated as follows: ^*^p < 0.1, ^**^p < 0.05, ^***^p < 0.01.

However, in subsequent conversion stages, the overall effect of Treatment × Creativity is negative, particularly in *Delayed Conversion* and *Num of Repeat Subscriptions*, where the interaction term coefficients are significantly negative (β = −0.65653, *p* < 0.01; β = −0.42434, *p* < 0.01, respectively). This finding suggests that while a longer free trial may increase initial user engagement, prolonged exposure to creativity-oriented functionalities during the trial period may weaken users' willingness to subscribe in the long run. These findings support the predictions of Hypothesis 2b and provide empirical validation for the theoretical framework proposed by Füller et al. (2008). The results reveal how product functionality influences the dynamic evolution of user “satisfaction” and “marginal utility” throughout the usage cycle. In particular, the highly exploratory nature of creativity-oriented functionalities may intensify the “cannibalization effect” or “usage fatigue effect” during an extended free trial period, ultimately reducing users' likelihood of immediately converting to a paid subscription after the trial.

### 5.5 The heterogeneous treatment effect

#### 5.5.1 Heterogeneous effects of users' economic background

According to the theories of quality signals (Kirmani and Rao, 2000; Wang and Özkan-Seely, 2018) and cannibalization effect (Cheng et al., 2015), the duration of free trials has a dual effect on user behavior. On the one hand, an extended trial period strengthens the quality assurance commitment, significantly enhancing users' trust in the product, thereby encouraging deeper exploration and use. On the other hand, an excessively long trial may lead to overuse of the product by users, which subsequently reduces the demand for future paid subscriptions, triggering a crowding-out effect. This section examines the heterogeneous impact of trial duration on user behavior across different economic contexts, as indicated by GDP ranking.

The research findings suggest that the level of economic development significantly moderates the effect of trial duration on user behavior at various stages of conversion, exhibiting a clear stratified effect. Specifically, as shown in Table 12, the interaction term GDP Ranking × Treatment demonstrates significant negative coefficients across all stages of user behavior, indicating that in economically less-developed regions (with lower GDP rankings), the positive effects of a longer trial period on user behavior are substantially diminished. More precisely, Column (1) of Table 12 shows that in regions with lower GDP rankings, a longer trial period actually weakens users' willingness to engage in the trial, reflecting the significant moderating effect of users' economic background on the quality signal effect. Results from Columns (2)–(4) further reveal that in economically less-developed areas, a longer trial period leads to a decrease in the conversion rate for paid subscriptions, which is closely linked to the crowding-out effect previously discussed.

**Table 12:** Impact of GDP ranking and group on subscription behaviors.

	**(1)**	**(2)**	**(3)**	**(4)**
**Variable**	**Free trial**	**Free trial**	**Delayed**	**Num of repeat**
GDP ranking	–0.00014^***^	–0.00004^***^	–0.00002^***^	–0.00007^***^
	[0.00012]	[0.00003]	[0.00002]	[0.00005]
Treatment	0.00201^***^	0.00076^**^	0.00114^***^	0.02120^***^
	[0.00070]	[0.00038]	[0.00031]	[0.00191]
GDP ranking × treatment	–0.00001^**^	–0.00001^**^	–0.00001^**^	–0.00016^***^
	[0.00001]	[0.00000]	[0.00000]	[0.00002]
Constant	0.02002^***^	0.00599^***^	0.00334^***^	0.00929^***^
	[0.00037]	[0.00020]	[0.00015]	[0.00025]
*N*	680,588	680,588	680,588	680,588
Adj.*R*^2^	0.0058	0.0025	0.0010	0.0023

Standard errors are shown in brackets. Significance levels are indicated as follows: ^*^p < 0.1, ^**^p < 0.05, ^***^p < 0.01.

Additionally, the interaction term GDP × VIP task downloads in Table 13 further explores how users' economic backgrounds and the number of tasks completed during the trial interact to modulate the crowding-out effect. The results indicate a significant negative correlation between this interaction and both Immediate conversion and repeat purchase stages, suggesting that in economically disadvantaged areas, as the number of completed VIP tasks increases, user demand is prematurely satisfied, thereby reducing conversion intentions at each stage. Therefore, it is recommended to limit the number of VIP task downloads or to implement phased unlocking features to sustain user demand and extend their interest in the product.

**Table 13:** Impact of GDP and VIP task downloads on subscription behaviors.

	**(1)**	**(2)**	**(3)**
**Variable**	**Immediate conversion**	**Delayed conversion**	**Num_Re_Sub**
GDP	–0.00005^***^	–0.00002^***^	–0.00011^***^
	[0.00000]	[0.00000]	[0.00000]
VIP task downloads	0.01086^***^	0.00451^**^	0.01958^***^
	[0.00323]	[0.00198]	[0.00639]
GDP × VIP task downloads	–0.00007^**^	–0.00001	–0.00011^**^
	[0.00003]	[0.00002]	[0.00006]
Constant	0.00605^***^	0.00360^***^	0.01524^***^
	[0.00017]	[0.00013]	[0.00058]
*N*	680,273	680,273	680,273
Adj. *R*^2^	0.0148	0.0071	0.0051

Standard errors are shown in brackets. Significance levels are indicated as follows: ^*^p < 0.1, ^**^p < 0.05, ^***^p < 0.01.

In summary, in economically developed regions, longer trial periods effectively transmit product quality signals, enhance user trust in the product's reliability and value, and foster deeper engagement and conversion. In contrast, in economically less-developed regions, prolonged trial periods may reduce subscription conversion rates by prematurely satisfying user demand, thereby exacerbating the crowding-out effect. To optimize trial outcomes in different economic contexts, it is recommended to shorten trial periods or restrict trial content in economically disadvantaged areas to reinforce the product's scarcity and immediate value, thus more effectively increasing users' purchase intentions.

#### 5.5.2 Heterogeneous effects of users' cultural background

Hofstede (1980) argues that Cultural IDV emphasizes personal interests and autonomy, whereas Cultural LTO focuses on long-term planning and the pursuit of future benefits. The findings of this study reveal that under extended trial periods, both cultural dimensions amplify users' decision-making effects related to trial conversion, but through fundamentally different mechanisms.

In IDV cultures, users are more likely to make autonomous decisions, basing their purchasing choices on personal needs and preferences rather than on societal expectations (Chen et al., 2017). A longer trial period gives these users sufficient time to independently evaluate the product's value, leading to a deeper understanding and more rational purchase decisions. Empirical results show that the interaction between **Individualism and extended trial periods (Cultural IDV** **×** **Treatment)** significantly boosts trial participation, paid conversion, repeat purchases, and total spending (see Table 14). In other words, longer trial durations enhance the product experience for individualistic users, improving both conversion rates and long-term retention.

**Table 14:** Impact of cultural IDV and treatment on subscription metrics.

	**(1)**	**(2)**	**(3)**	**(4)**
**Variable**	**Free trial**	**Immediate**	**Delayed**	**Num of repeat**
Cultural IDV	0.00022^***^	0.00008^***^	0.00003^***^	0.00011^***^
	[0.00001]	[0.00000]	[0.00000]	[0.00000]
Treatment	–0.00028	–0.00039*	–0.00009	–0.00319^***^
	[0.00047]	[0.00023]	[0.00021]	[0.00123]
Cultural IDV × treatment	0.00003^**^	0.00001^*^	0.00002^***^	0.00027^***^
	[0.00001]	[0.00001]	[0.00001]	[0.00004]
Constant	–0.00025	–0.00087^***^	0.00003	–0.00088^***^
	[0.00024]	[0.00012]	[0.00010]	[0.00016]
*N*	679,506	679,506	679,506	679,506
Adj. *R*^2^	0.0045	0.0022	0.0008	0.0020

Standard errors are shown in brackets. Significance levels are indicated as follows: ^*^p < 0.1, ^**^p < 0.05, ^***^p < 0.01.

By contrast, in cultures with a strong **LTO**, which prioritize delayed gratification, long-term investment, and future rewards, extending the trial period does not facilitate user conversion. Instead, it triggers a systematic decline throughout the entire conversion process, driven by opportunity cost considerations, decision-making delays, and resource optimization behaviors (see Table 15). Specifically, LTO users are less likely to adopt a free trial in the first place, as they prefer to wait for external validation, such as peer reviews or broader market feedback, before engaging. During the trial period, a longer window reduces the perceived urgency to evaluate and decide, leading users to postpone or even overlook subscription decisions, thereby weakening immediate conversion outcomes (Dhar, 1997). After the trial, LTO users tend to continue optimizing their resources by favoring more cost-effective alternatives, resulting in lower rates of delayed conversion and repurchase. Overall, these patterns suggest that in LTO-oriented markets, shortening the trial period may be more effective in enhancing decision-making urgency and improving overall conversion rates. Therefore, in markets characterized by strong **Long-Term Orientation**, appropriately shortening the trial duration can help instill a greater sense of urgency, encouraging users to engage in faster evaluation and purchasing decisions, thereby optimizing trial conversion rates and strengthening long-term user retention.

**Table 15:** Impact of cultural LTO and treatment on subscription metrics.

	**(1)**	**(2)**	**(3)**	**(4)**
**Variable**	**Free trial adoption**	**Immediate conversion**	**Delayed conversion**	**Num_Re_Sub**
Cultural LTO	–0.00013^***^	–0.00003^***^	–0.00001^***^	–0.00005^***^
	[0.00000]	[0.00000]	[0.00000]	[0.00000]
Treatment	0.00185^***^	0.00018	0.00104^***^	0.01375^***^
	[0.00054]	[0.00027]	[0.00024]	[0.00142]
Cultural LTO × treatment	–0.00002^**^	–0.00000	–0.00001^**^	–0.00014^***^
	[0.00001]	[0.00000]	[0.00000]	[0.00002]
Constant	0.01397^***^	0.00370^***^	0.00195^***^	0.00560^***^
	[0.00028]	[0.00015]	[0.00011]	[0.00018]
*N*	679,506	679,506	679,506	679,506
Adj. *R*^2^	0.0012	0.0003	0.0001	0.0011

Standard errors are shown in brackets. Significance levels are indicated as follows: ^*^p < 0.1, ^**^p < 0.05, ^***^p < 0.01.

## 6 Robustness checks

To rigorously validate the robustness of our findings, we conducted two additional robustness tests.

First, we narrowed the experimental period from July 18, 2022 to August 8, 2022, to a shorter time frame from July 28, 2022 to August 1, 2022, retaining only users who were randomly assigned to the experimental group within this five-day period, while maintaining the observation period at 2 years. By refining the temporal scope, we aimed to assess the consistency of user behavioral outcomes over a more focused and condensed timeframe, thereby mitigating potential confounding effects related to temporal variations in the original study window. The results, presented in Appendix Tables A1, A2, are consistent with those obtained under the original experimental framework (Tables 4, 5), thereby reinforcing the validity of our conclusions.

Second, to further verify the robustness of our results, we supplemented our Ordinary Least Squares (OLS) regression analysis with a Logit model to account for the binary nature of the dependent variables. The Logit regression model allows for an examination of factors influencing binary outcomes and facilitates the establishment of a causal relationship between free trial duration and user conversion (Hoetker et al., 2007). As shown in Tables A3, A4, the primary effects observed in the Logit regression closely align with those from the OLS regression.

Specifically, in Table A3, the coefficient of the Treatment variable for Free Trial Adoption and Delayed Conversion is positive and statistically significant at the *p* < 0.05 level, indicating a significant impact on user conversion at these two stages. Moreover, the Treatment variable does not exhibit a significant effect on Immediate Conversion, a finding consistent with the OLS regression results. Similarly, in Table A4, the Treatment variable remains positively significant for all long-term conversion outcome variables, further corroborating the findings from the OLS regression.

These robustness checks provide additional empirical evidence supporting the reliability and generalizability of our results. By adjusting the sample period and exploring different subsets of the dataset, we further validate the stability of our conclusions, ensuring that our findings are not overly sensitive to variations within the dataset.

## 7 Conclusion and implications

### 7.1 Key findings and managerial implications

This study makes a significant contribution to optimizing free trial strategies within the Freemium model for SaaS products. We propose a three-stage user conversion framework based on the user conversion path and systematically quantify the impact of trial duration on overall subscription rates and each conversion stage, filling a critical gap in the existing literature. The research not only enriches the theoretical framework but also provides practical guidance for companies to optimize trial design in real-world applications.

From a managerial perspective, extending the free trial period can effectively enhance new users' trust in product quality and promote deeper exploration and engagement with core functionalities. Although the extension of trial duration may not directly improve immediate conversion rates due to the offsetting effects of the learning effect and demand cannibalization, its long-term positive effects gradually become apparent. Extended trials significantly increase opportunities for users to experience premium features, foster stronger product dependency, and thereby enhance reactivation rates and ultimate subscription conversion rates in subsequent promotional stages. Corporate operational data validate these findings: after implementing a 7-day free trial strategy, user engagement improved significantly, and the long-term growth of users' lifetime value (LTV) was sustainably boosted. This suggests that although extending the trial period may temporarily suppress immediate subscriptions, it strengthens user stickiness and future conversion potential, ultimately driving more sustainable platform growth.

Further analysis reveals that combining extended trials with feature-driven promotional strategies outperforms price-driven promotions. For users experiencing longer trials, promotions introducing new features are more effective in enhancing long-term retention and subscription intentions; by contrast, users with shorter trials respond more sensitively to price discount promotions. In addition, product functionality plays a significant moderating role in the relationship between trial duration and conversion. For creative features emphasizing exploratory experiences, extended trials may decrease conversion rates due to demand saturation. In contrast, for task-oriented functionalities focused on accomplishing specific goals, longer trial periods significantly enhance users' willingness to pay. This finding aligns with the conclusions of Füller et al. (2008) and highlights the critical role of product attributes in influencing user purchase decisions. Therefore, when designing trial periods, firms should comprehensively consider users' experiential paths, feature usage frequency, and psychological expectations to develop targeted optimization strategies.

Meanwhile, based on user data from nearly 190 countries, this study reveals significant heterogeneity in user responses to trial duration strategies across different cultural and economic contexts. On the one hand, in markets characterized by high Individualism, extending the trial period effectively increases conversion rates, as users are more inclined to engage in independent exploration and make purchasing decisions based on personal experience. In contrast, in markets with high Long-Term Orientation, longer trial periods tend to suppress subscription intentions due to longer decision-making cycles and weaker immediate value perceptions. On the other hand, economic development levels also significantly moderate the effects of trial duration. In regions with lower GDP rankings (economically less-developed markets), the positive impact of extended trials is substantially weakened, and a crowding-out effect emerges, where users fulfill their core needs during the trial period and subsequently reduce their willingness to subscribe. Overall, these findings suggest that firms should flexibly tailor trial duration strategies according to the cultural and economic characteristics of target markets to maximize conversion rates and long-term revenue potential.

### 7.2 Practical implications

This study not only provides systematic managerial guidance for SaaS firms but also offers actionable insights for practical operations. First, companies should adopt a three-stage user conversion framework when designing trial mechanisms, systematically considering the synergistic effects across immediate conversion, delayed conversion, and long-term retention, rather than focusing on a single conversion metric in isolation.

Second, firms should flexibly adjust trial durations based on the nature of product features. For creative and exploratory features, moderately shortening the trial period can help mitigate demand cannibalization. In contrast, for task-oriented features with strong necessity in usage scenarios, extending the trial period effectively enhances user stickiness and subsequent paid conversion.

In terms of promotional strategies, companies need to tailor their approaches based on users' trial duration experiences. For users who have experienced longer trials, feature-driven promotions (e.g., new feature launches, upgrade announcements) should be prioritized to reinforce the perceived added value. For users with shorter trial experiences, price-based promotions (e.g., limited-time discounts) can be employed to trigger immediate subscription decisions.

More importantly, in the process of global market expansion, firms should dynamically adjust free trial designs by fully considering the cultural and economic characteristics of different regions. In highly individualistic markets, extending the trial period helps stimulate user-driven exploration and enhances subscription conversion; whereas in long-term-oriented cultures, appropriately shortening the trial period can strengthen decision-making urgency and improve overall conversion efficiency. Simultaneously, in economically developed regions, firms can leverage longer trial periods to deepen user experience and foster long-term relationships; while in less-developed markets, it is advisable to shorten the trial period or restrict the scope of free features to prevent demand saturation and premature fulfillment. By dynamically optimizing trial mechanisms based on cultural and economic differences, firms can more precisely stimulate user demand, enhance conversion efficiency, and achieve more sustainable growth in global expansion efforts.

In summary, through theoretical innovation and empirical validation, this study proposes a systematic framework and practical strategies for optimizing free trial mechanisms within the Freemium model. It not only enriches the academic research landscape but also provides implementable guidance for enterprises aiming to improve conversion rates and enhance LTV in real-world operations.

### 7.3 Limitations and future research directions

Despite its contributions, this study has several limitations. First, it focuses on short-duration trials for utility-based SaaS products (e.g., 3-day, 7-day, or 14-day trials). However, products with greater complexity and higher learning costs–such as enterprise software or professional tools–may exhibit distinct user behavior patterns. In the case of longer trial periods (e.g., one month or more), users' learning curves, perceived value, and subscription decisions may be driven by different factors. Future research should further investigate how varying trial durations influence user learning effects and conversion pathways to provide a more comprehensive understanding of causal mechanisms.

Second, the external generalizability of the findings remains to be validated. While this study primarily examines SaaS products, whether its conclusions apply to other industries that employ free trial models warrants further examination. Different product categories, such as physical goods or content-based services (e.g., online education and streaming platforms), may involve distinct user decision-making processes. Future studies should explore the applicability of these findings across a broader range of contexts.

Finally, the lack of detailed demographic data at the individual level limits this study's ability to precisely capture user heterogeneity effects. Factors such as users' economic status, occupational background, cultural background, and purchasing behavior may significantly influence trial engagement and conversion pathways. Future research should integrate more granular user profiling to refine trial strategies for different market segments, thereby enhancing both explanatory power and practical relevance.

## Data Availability

The datasets presented in this article are not readily available because the data used in this study were provided by corporate partners and contain proprietary business information and user privacy details. According to confidentiality agreements, the raw data cannot be made publicly available. However, upon reasonable request and subject to appropriate anonymization and approval by the data provider, the data can be made accessible to editors, reviewers, or other authorized parties for the purpose of review and verification. Requests to access the datasets should be directed to the corresponding author.

## Footnotes

1 The experiment utilized a hash function to perform pseudo-random assignment based on unique user identifiers (e.g., user IDs, device IDs, IP addresses), ensuring fair allocation and maintaining data stability.

2 For quantification in the randomization checks, we assigned values to the five registration channels as follows: apple = 1, email = 2, facebook = 3, fotor = 4, google = 5.

3 We assigned values to the five traffic sources Assigned as follows: Organic Traffic = 1, Direct Traffic = 2, Referral Traffic = 3, Social Media Traffic = 4, Paid Traffic = 5, NULL = 0.

4 These proportions were established prior to the experiment, considering uncertainties and business impact. Following the demonstration of the 7-day trial's superior effectiveness, it is now implemented universally.
