# Correction: Longer or shorter? A large-scale randomized field experiment on the impact of free trial duration on sustainable user conversion in the Freemium model

**DOI:** 10.3389/fpsyg.2025.1732439

**Published:** 2025-11-10

**Authors:** Ling Zhang, Jiang Duan

**Affiliations:** 1 School of Computing and Artificial Intelligence, Southwestern University of Finance and Economics, Chengdu, China; 2 Chengdu Agricultural College, Chengdu, China

**Keywords:** Freemium model, free trial duration, user conversion, field experiment, behavioral operations

In the published article, an additional affiliation was erroneously omitted for author Ling Zhang. The previous information read:

“Ling Zhang and Jiang Duan^*^

School of Computing and Artificial Intelligence, Southwestern University of Finance and Economics, Chengdu, China”

The correct affiliation information appears below:

“Ling Zhang^1, 2^ and Jiang Duan^1^^*^

^1^School of Computing and Artificial Intelligence, Southwestern University of Finance and Economics, Chengdu, China^2^Chengdu Agricultural College, Chengdu, China”

The original version of this article has been updated.

