# Supplementary material for: Longer or shorter? A large-scale randomized field experiment on the impact of free trial duration on sustainable user conversion in the Freemium model
Source: Front Psychol. 2025 Jun 18;16:1568868. doi: 10.3389/fpsyg.2025.1568868 (PMC12217587; doi:10.3389/fpsyg.2025.1568868)
Supplement: Supplementary file 1 [file Data_Sheet_1.pdf]

Appendix

Table A.1 Impact of Treatment on Free Trial Adoption and Conversion

Variable	(1) Free Trial Adoption	(2) Immediate Conversion	(3) Delayed Conversion
Treatment	0.00115***	0.00026	0.00045***
	[0.00037]	[0.00023]	[0.00016]
Constant	0.00755***	0.00328***	0.00034***
	[0.00016]	[0.00017]	[0.00008]
N	153890	153890	153890
adj. R^2	0.0011	0.0007	0.0016

Note: Standard errors are shown in brackets. Significance levels are indicated as follows: $^{*}p < 0.1$, $^{**}p < 0.05$, $^{***}p < 0.01$.

Table A.2 Impact of Treatment on Various Subscription Metrics

Variable	(1) Overall Subscription	(2) Cumulative Spending	(3) Num_Re_Sub	(4) CA_Sub
Treatment	0.00081***	0.08751***	0.00889***	0.00320***
	[0.00027]	[0.03214]	[0.00066]	[0.00048]
Constant	0.00316***	0.17843**	0.00455***	0.01073***
	[0.00021]	[0.00554]	[0.00019]	[0.00028]
N	153890	153890	153890	153890
adj. R^2	0.000341	0.000436	0.004816	0.000623

Note: Standard errors are shown in brackets. Significance levels are indicated as follows: $^{*}p < 0.1$, $^{**}p < 0.05$, $^{***}p < 0.01$.

Table A.3 Impact of Treatment on Free Trial Metrics

Variable	(1) Free trial Adoption	(2) Immediate Conversion	(3) Delayed Conversion
Treatment	0.10664***	0.07102	0.35556***
	[0.02781]	[0.05476]	[0.06235]
Constant	-4.75254***	-6.09686***	-6.54489***
	[0.01568]	[0.03052]	[0.03815]
N	680588	680588	680588
adj. R^2	0.0324	0.0022	0.00597

Note: Standard errors are shown in brackets. Significance levels are indicated as follows: $^{*}p < 0.1$, $^{**}p < 0.05$, $^{***}p < 0.01$.

Table A.4 Impact of Treatment on Various Subscription Metrics

Variable	(1) Overall Subscription	(2) Cumulative Spending	(3) Num_Re_Sub	(4) CA_Sub
Treatment	0.00079***	0.04645***	0.00796***	0.00227***
	[0.00017]	[0.01087]	[0.00064]	[0.00038]
Constant	0.00367***	0.17837***	0.00366***	0.01033***
	[0.00009]	[0.00524]	[0.00009]	[0.00019]
N	680588	680588	680588	680588
adj. R^2	0.0012	0.0011	0.0103	0.0025

Note: Standard errors are shown in brackets. Significance levels are indicated as follows: $^{*}p < 0.1$, $^{**}p < 0.05$, $^{***}p < 0.01$.
